# Methodological challenges and new perspectives of shifting vegetation phenology in eddy covariance data

**DOI:** 10.1038/s41598-023-41048-x

**Published:** 2023-08-24

**Authors:** Annu Panwar, Mirco Migliavacca, Jacob A. Nelson, José Cortés, Ana Bastos, Matthias Forkel, Alexander J. Winkler

**Affiliations:** 1 Department of Biogeochemical Integration, Max Planck Institute for Biogeochemistry, Hans-Knöll-Straße 10, 07745 Jena, Germany; 2 European Commission, Joint Research Centre (JRC), Ispra, Lombardia, Italy; 3 TUD Dresden University of Technology, Faculty of Environmental Sciences, Dresden, Germany

**Keywords:** Climate-change impacts, Environmental impact, Carbon cycle

## Abstract

While numerous studies report shifts in vegetation phenology, in this regard eddy covariance (EC) data, despite its continuous high-frequency observations, still requires further exploration. Furthermore, there is no general consensus on optimal methodologies for data smoothing and extracting phenological transition dates (PTDs). Here, we revisit existing methodologies and present new prospects to investigate phenological changes in gross primary productivity (GPP) from EC measurements. First, we present a smoothing technique of GPP time series through the derivative of its smoothed annual cumulative sum. Second, we calculate PTDs and their trends from a commonly used threshold method that identifies days with a fixed percentage of the annual maximum GPP. A systematic analysis is performed for various thresholds ranging from 0.1 to 0.7. Lastly, we examine the relation of PTDs trends to trends in GPP across the years on a weekly basis. Results from 47 EC sites with long time series (> 10 years) show that advancing trends in start of season (SOS) are strongest at lower thresholds but for the end of season (EOS) at higher thresholds. Moreover, the trends are variable at different thresholds for individual vegetation types and individual sites, outlining reasonable concerns on using a single threshold value. Relationship of trends in PTDs and weekly GPP reveal association of advanced SOS and delayed EOS to increase in immediate primary productivity, but not to the trends in overall seasonal productivity. Drawing on these analyses, we emphasise on abstaining from subjective choices and investigating relationship of PTDs trend to finer temporal trends of GPP. Our study examines existing methodological challenges and presents approaches that optimize the use of EC data in identifying vegetation phenological changes and their relation to carbon uptake.

## Introduction

Vegetation phenology is the periodic pattern of plant life cycle such as leaf unfolding and leaf senescence. Phenological records spanning over 19th and early 20th century were mainly applied on the local scale in the fields of forestry, agriculture and human health. Few decades later, phenological observatories and remote sensing products expanded worldwide, and studies reported strong response of vegetation phenology to climate change^1,2^. Around the 1980s, the eddy covariance (EC) technique emerged as a tool to monitor net carbon exchange in the ecosystem level^3^. Phenology inferred from EC techniques are extensively used for remote sensing product validation^4,5^. So far, remote sensing has contributed substantially in the field of land surface phenology^6^. However, there remains high uncertainty in elucidating the mechanistic drivers of phenological changes using remote sensing products^7^. In this context, frequent observations of carbon, heat and water fluxes provided by EC data offers emerging opportunities to understand the mechanisms driving phenological trends and their feedback to climate change^8^. The application of EC data for detecting changes in phenology is still in its earlier stage. Present EC sites offer relatively longer time series that are required to derive reliable trends. Even so, in order to leverage the potential of EC data, the associated methodological challenges must be thoroughly addressed.

While field observations and remote sensing products across Europe^9^, China^10^, and North America^11^ report an earlier start of spring and delayed onset of autumn in connection to global warming. Few studies on phenological trends at EC sites report contradictory findings. For example, a study by^12^ shows no trends in 56 EC sites, while another by^13^ used 47 EC sites and revealed an earlier spring and autumn in forests and a delay in grasslands. These opposing results can be rooted in different methodologies used to smooth and extract PTDs from the high variability of EC time series. For example, while^12^ used a moving average filter,^13^ used a Savitzky-Golay filter to smooth the seasonal cycle of daily GPP data. For PTDs calculation both studies used threshold approach, that prescribe a certain threshold between the minimum and maximum seasonal cycle to indicate that vegetation has reached a specific phenological state. The two studies used different thresholds for PTDs, where^12^ employed 15% of the multi-year daily GPP maximum value as threshold whereas the second study^13^ used 25% threshold.

Several smoothing and phenology detection methods are discussed and compared in the field of remote sensing^14,15^. Subjectivity on selection of smoothing function has been previously acknowledged with a convincing notion that no single smoothing method performs better than the other. Software packages like TIMESAT^16^ allow the user to determine PTDs from the time series of normalized difference vegetation index (NDVI) based on different smoothing functions. Differences of 20 to 50 days in estimation of SOS and the length of the growing season (GSL = EOS − SOS) were recorded when Savitzky-Golay and double logistic smoothing functions^17^ were used in TIMESAT. For similar reasons, remote sensing oriented studies recommend exploring different smoothing methods accompanied by visual inspection. Smoothing parameters might need to be adjusted for specific plant functional types (PFTs), surface characteristics and vegetation indices^18^. While insights gained on smoothing methods in reference to satellite data are useful, they might not be always directly transferable to EC data due to the differences in spatial coverage and temporal frequencies. Furthermore, continuous carbon fluxes estimated by EC technique, such as Gross primary productivity (GPP), have direct association to vegetation activity^8^, whereas vegetation indices retrieved from remote sensing might not^19^.

**Figure 1:**
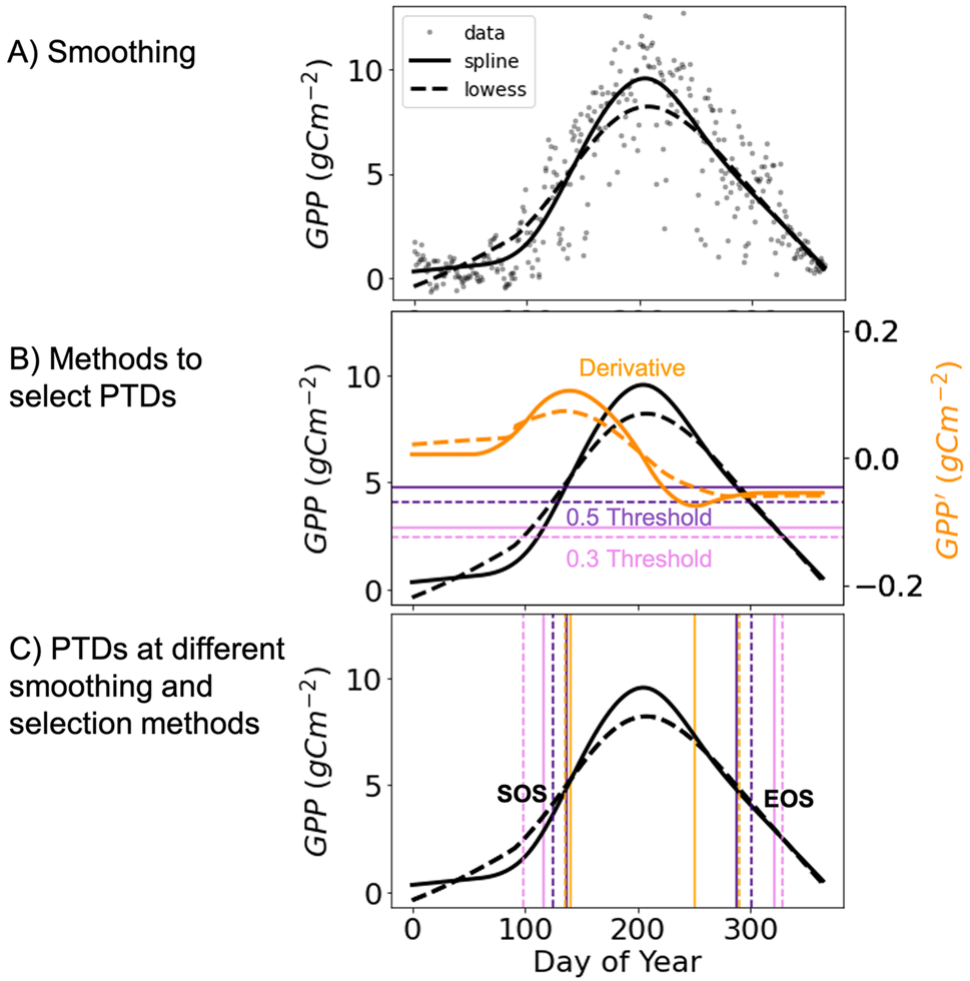
A schematic diagram of different smoothing functions and Phenological Transition Dates (PTDs) selection methodologies. Data: GPP values for year 2001 at an evergreen needleleaf forest, eddy covariance site at Tharandt Forest (DE-Tha), Germany. (**A**) The smoothing of raw data (dotted points) using spline (7 knots) and lowess (0.5 smoothing fraction) methods. (**B**) Depiction of the threshold and first derivative methods to select the start (SOS) and end of season (EOS) for spline and lowess smoothed GPP. (**C**) SOS and EOS obtained from different threshold values and first derivative method. The PTDs also differ for spline and lowess smoothed GPP time series.

Gross primary productivity (GPP) is a measure of the overall rate of carbon fixation through photosynthesis. However, GPP estimates based on models and proxies are prone to noise due to the stochastic nature of turbulence and uncertainties in partitioning algorithms^20^. To obtain phenology from GPP, it is necessary to smooth the daily fluctuations and noise in the signal. It is important to note that different smoothing functions can yield different outcomes. Figure 1A illustrates this by comparing smoothed GPP obtained from two commonly used spline and lowess smoothing functions. The difference between the two smoothing functions is most noticeable in the spring and around the peak of the season. It should be acknowledged that the choice of smoothing function is influenced by parameters that determine the fit to the data. For example, spline is a piecewise regression method that is sensitive to the number of segments or knots provided by the user, while Lowess smoothing is a weighted local linear fit controlled by the defined fraction of data used in each fit. Additionally, different methodologies define PTDs differently. The threshold method compares the smoothed vegetation index to a fixed percentage of its annual maximum, with the days passing this threshold before and after the annual maximum defined as the start and end of the season, respectively. On the other hand, the derivative method looks for the maximum and minimum values of the first derivative of smoothed GPP, which are then inferred as the start and end of the season, respectively. Figure 1B shows the positions of the 0.3 (30% of maximum GPP) and 0.5 (50% of maximum GPP) thresholds, and Fig. 1C shows the start of season (SOS) and end of season (EOS) obtained at these thresholds from spline and lowess smoothed GPP. Similarly, SOS and EOS obtained from the first derivative approach show slight differences for different smoothing functions.

Accounting for the influence of different smoothing and PTDs selection methods is crucial for trend analysis. Trends might vary when PTDs are obtained at different thresholds or it could be that an optimal threshold best captures the phenological changes of interest^21^. Previous studies tend to use a threshold value of 0.2 that occur around the timing of early bud bursting and leaf unfolding during spring. A higher 0.5 threshold is also a typical choice for capturing the rapid change in leaf greenness^22^. Even so, the threshold values might represent different stages of vegetation activity for different plant functional types. For instance, satellite based spring phenology were found highly correlated to ground phenology of broadleaf trees at 0.75 threshold, but at much lower 0.2 threshold for the understory plants^23^. Moreover, different vegetation indices obtained from remote sensing can produce dissimilar PTDs at a single threshold. As shown previously, spring onset calculated at the 0.5 threshold using the normalized difference vegetation index was closer to the date of GPP at 0.1 threshold. But spring onset based on leaf area index was found closer to the date of GPP at 0.2 threshold^24^. Different footprints of satellite and EC sites to some extent cause this mismatch. Although not yet explicitly confirmed, choices of thresholds might also explain observed discrepancies of phenological trend analysis^25^. To our knowledge no previous study has investigated trends of PTDs at different thresholds at ecosystem level using EC data. In this study we revisit derivative and threshold methods used to determine PTDs using GPP time series and present new perspectives on phenological trend analysis utilizing EC data.

Existing studies specifically addressed the trends of seasonal^26^ and annual flux integral of GPP in model or remote sensing data^27^. Few studies show the monthly scale GPP and its response to climate factors^28,29^. In this context, the daily availability of EC data is not utilized to its full potential, and in our view trend analysis of finer temporal scale captures valuable information on the dynamic of ecosystem productivity and their relation to PTDs shifts. By relating trends in PTDs to weekly trends in GPP, we evaluate how the shift in SOS and EOS impact the subsequent and overall annual productivity.

In this study, we present a new simple smoothing method called integral smoothing, which derives the smoothed time series of GPP through the derivative of its smoothed annual cumulative sum. We demonstrate that integral smoothing is more robust to user-defined smoothing parameters compared to directly smoothing the raw GPP signals. Additionally, we investigate the differences in PTDs and their trends when calculated at various threshold values. We identify sites with high variability in PTD trends among different thresholds. Furthermore, we highlight the importance of examining weekly trends of GPP throughout the year, as it provides additional mechanistic insights into phenological changes, beyond solely analyzing trends in PTDs.

## Data and methods

### Data

For this study eddy covariance data was aggregated from the FLUXNET 2015^30^ , ICOS warm winter 2020^31^ and AmeriFlux FLUXNET, all of which were processed using the OneFlux processing pipeline^30^. The site information is summarized in a table in the data availability section. For general analysis of PTDs total number of 83 EC sites^30^ are used. For the trend analysis, 47 EC sites that have more than 10 years of quality data are selected. To determine vegetation phonological transition dates we use the time series of GPP. It is to be pointed that eddy covariance towers do not measure GPP directly, it is obtained from the partitioning of net ecosystem exchange (NEE). In this study we use daily estimates of GPP obtained from the night-time partitioning method^32^ where the night-time data are used to parameterize a respiration model. This method is based on the assumption that during night-time, plants primarily undergo respiration and there is no assimilation of carbon dioxide. Since GPP is modelled from NEE, so it is expected that missing or poor quality of GPP data originates from the poor quality of NEE observations. Based on these reasoning, in our work the quality of GPP data is inferred from the quality flag of NEE. Years with more than 70% of days with good quality NEE (original or good quality gap filled from the OneFlux NEE_QC) data are included in our analysis. For the trend related analysis, results are aggregated for specific PFTs. There are 11 cropland (CRO), 8 grassland (GRA), 4 mixed forest (MF), 15 evergreen needle-leaf forest (ENF) and 9 deciduous broad-leaf forest (DBF) EC sites used for phenological trend analysis.

### Smoothing

**Figure 2:**
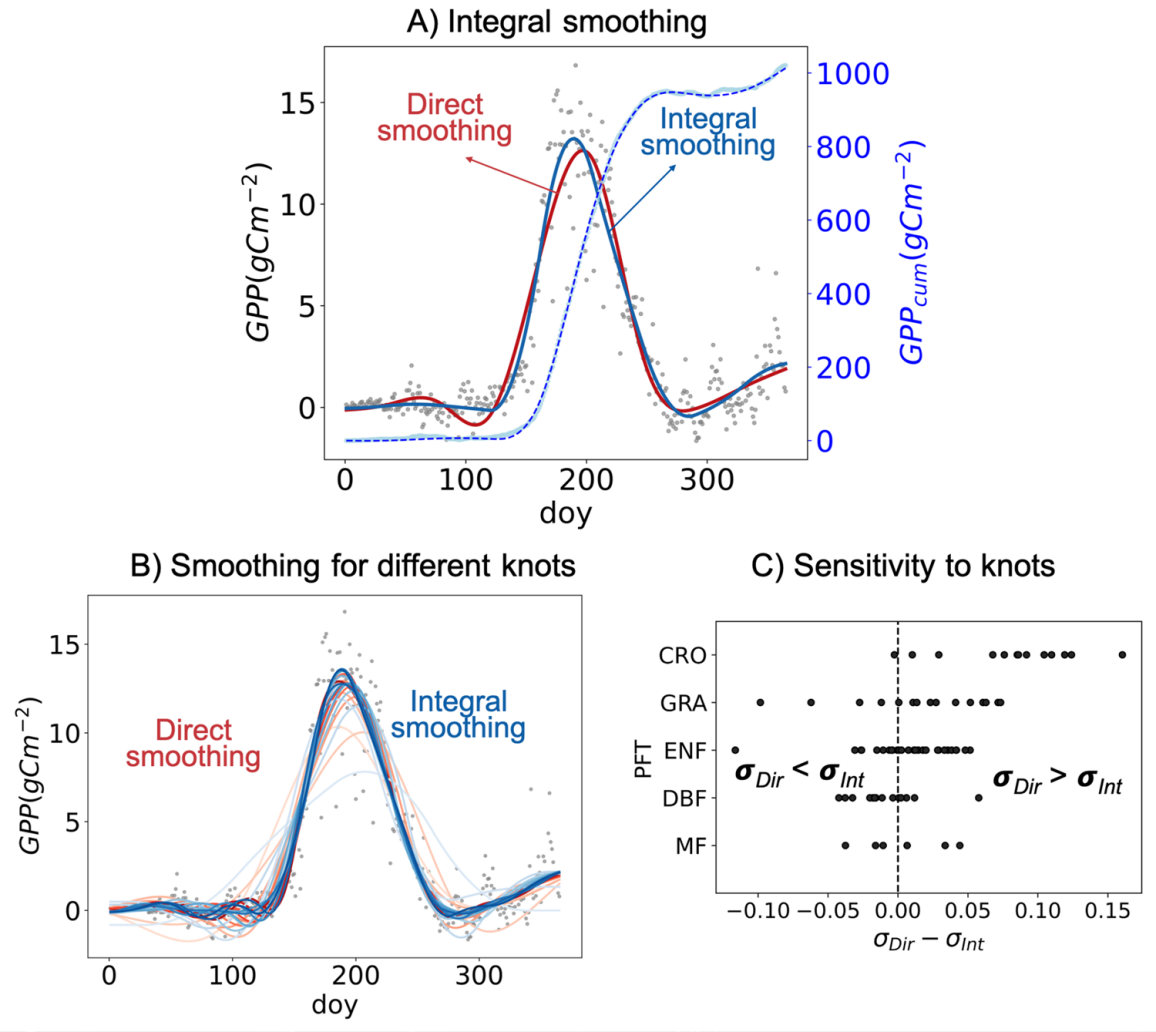
(**A**) An illustration of Integral smoothing that obtains smoothed GPP through the derivative of its annual cumulative time series. Data is smoothed with spline function (10 knots) (**B**) Smoothed GPP obtained from direct and integral smoothing method using spline function at different knots (5–18). Data for A and B: year 2001, US-Bo1: Bondville eddy covariance site, Grassland. (**C**) Sensitivity of direct and integral smoothing to perturbations of the number of knots in 83 EC sites belonging to different PFTs. The sensitivity is obtained as the mean standard deviation ($\sigma$) of smoothed time-series for different knots (5–18). Higher standard deviation depicting high sensitivity to knots. The x-axis shows the difference between the standard deviation calculate for direct ($\sigma _{Dir}$) and integral ($\sigma _{Int}$) smoothing.

Here we propose integral smoothing for GPP time series: rather than smoothing GPP time series directly, we first calculate its cumulative sum for the calendar year and smooth its integral. In order to produce continuous multi-year time series along with the year in consideration the last 30 days of the preceding and first 30 days of the following calendar year are also used. As apparent in Fig. 2A, the cumulative sum of GPP time series is continuous and has higher signal to noise ratio than the original GPP time series, making the smoothing more straightforward than the former. The first step of integral smoothing is to smooth the cumulative sum of GPP, shown in blue dashed curve. For smoothing we use spline function with 10 number of knots. Lastly, the smoothed GPP (blue curve) is obtained from the first derivative of the smoothed time series of the GPP cumulative curve. Likewise, integral smoothing is performed for each calendar year. The smoothed GPP for the year in consideration is then obtained by removing the extra 60 days of the preceding and the following years. For the first/last year of the GPP time series, the last/first 30 days of the same year are used to obtain the extra 60 days that the smoothing filter requires.

Figure 2A shows that integral smoothing produces a slightly different fit than the direct smoothing of GPP. In this case, their differences are more evident around the start of season and near the peak of season, when the signals are weaker than the noise. To further assess the sensitivity of direct and integral smoothing to user defined smoothing parameters Fig. 2B show smoothed GPP for different knots number ranging from 5 to 18. It is apparent that smoothed GPP obtained from lower and higher knots are substantially different for direct and integral smoothing. To quantify the sensitivity of each smoothing to knot numbers, based on 83 EC sites we then calculate the standard deviation ($\sigma$) between the smoothed GPP time-series calculated for different knots. Figure 2C shows the differences of $\sigma$ between direct and integral smoothing for all the sites of different PFTs. For most of the sites belonging to CRO, GRA and ENF, $\sigma$ for direct smoothing is higher indicating greater differences in smoothed GPP when different number of knots are used. Overall, Fig. 2C confirms the high objectivity (through low $\sigma$) of integral smoothing to number of spline smoothing knots over the traditional direct smoothing method.

Smoothing of vegetation signals is the primary step to obtain phenological transition events. Hereafter, our analysis uses integral smoothing to obtain smoothed GPP time series in EC data. For consistency, in all the cases the number of knots used for integral smoothing is set to 10. Based on visual inspection we deduce the suitability of using 10 knots for all the EC sites. As already demonstrated in Fig. 2, integral smoothing is more robust towards the number of knots, therefore results might not perturb to a greater extent if knots are changed for instance from 9 to 12. Based on our analysis in Fig. 2 it can be ensured that the influence of number of knots is weaker for integral smoothing than the direct smoothing. We suggest to not use a lower number of knots (< 6) given it can produce over-smoothing. Conversely, higher knots number can result in over-fitting and incorporate too much variability that is not appropriate for calculating phenological transition dates.

### Calculation of phenological transition dates and their trends

In this study we calculate PTDs using two highly cited threshold and derivative methods. For the threshold method, the annual GPP is first normalized (0 to 1) using the minimum-maximum approach. The day of year corresponding to a given threshold of normalized GPP curve is then identified, the first point is defined as SOS and the second as EOS. PTDs are calculated for every 0.05 increment in the threshold values ranging from 0.1 to 0.7. In addition to threshold methods, PTDs are also calculated from the first derivative of smoothed GPP cycle. Derivative method is viewed as an ecologically meaningful approach that is capable of handling multiple growth cycles^33^. Day of year with maximum value of first derivative are assigned as SOS and day with minimum as EOS. It should be noted that derivative method is sensitive to smoothing of GPP. One of our secondary objective of using derivative method is to examine how it compares to PTDs calculated from different thresholds.

For some sites and years, the annual GPP curve is not symmetric and the threshold might not be crossed twice in the same year. In such cases the start/end of season fall in the previous/next year. This technique is effective for ecosystem where the entire seasonal cycle does not occur in the same calendar year. Moreover, cases are identified when the threshold is crossed more than one day before or after the peak of season, a common observation specially in autumn phenology. For such cases, crossings closest to the peak of season are assigned as PTDs. For ecosystem with two growing season, the peak is assigned to the most productive growing season, meaning single SOS and EOS for one calender year. In principle our approach works well for EC sites in the southern hemisphere, since our method can look for SOS and EOS in the previous and next years. More information on our method is provided in a Python package called EasyPhenology^34^.

Trend analysis of PTDs is performed for 47 EC sites that have more than 10 years of quality data. Trends are calculated using the Theil-Sen estimator, which is resistant to the outliers and tends to lead accurate confidence intervals^25^. The Theil-Sen estimator is the median of all possible pairwise slopes in a time-series, defined as

1 $$\begin{aligned} PTD_{Trend} = median \left( \sum _{i=1}^{n-1} \sum _{j=i+1}^{n} \frac{y_j - y_i}{j-i} \right) \end{aligned}$$

Here, *y* represents PTD, and *n* is the total number of years.

SOS, EOS and growing season length (GSL = EOS − SOS) and their trends are calculated for different threshold values. Please note, the trend in GSL is not the difference of the trends in EOS and trends in SOS, but is the trends of the difference between EOS and SOS. One of our primary objectives is to determine if the trends in PTDs vary significantly when PTDs are calculated from different threshold values. To quantify this we calculate the standard deviation among trends.

## Results

### Phenological transition dates and thier trends at different thresholds

**Figure 3:**
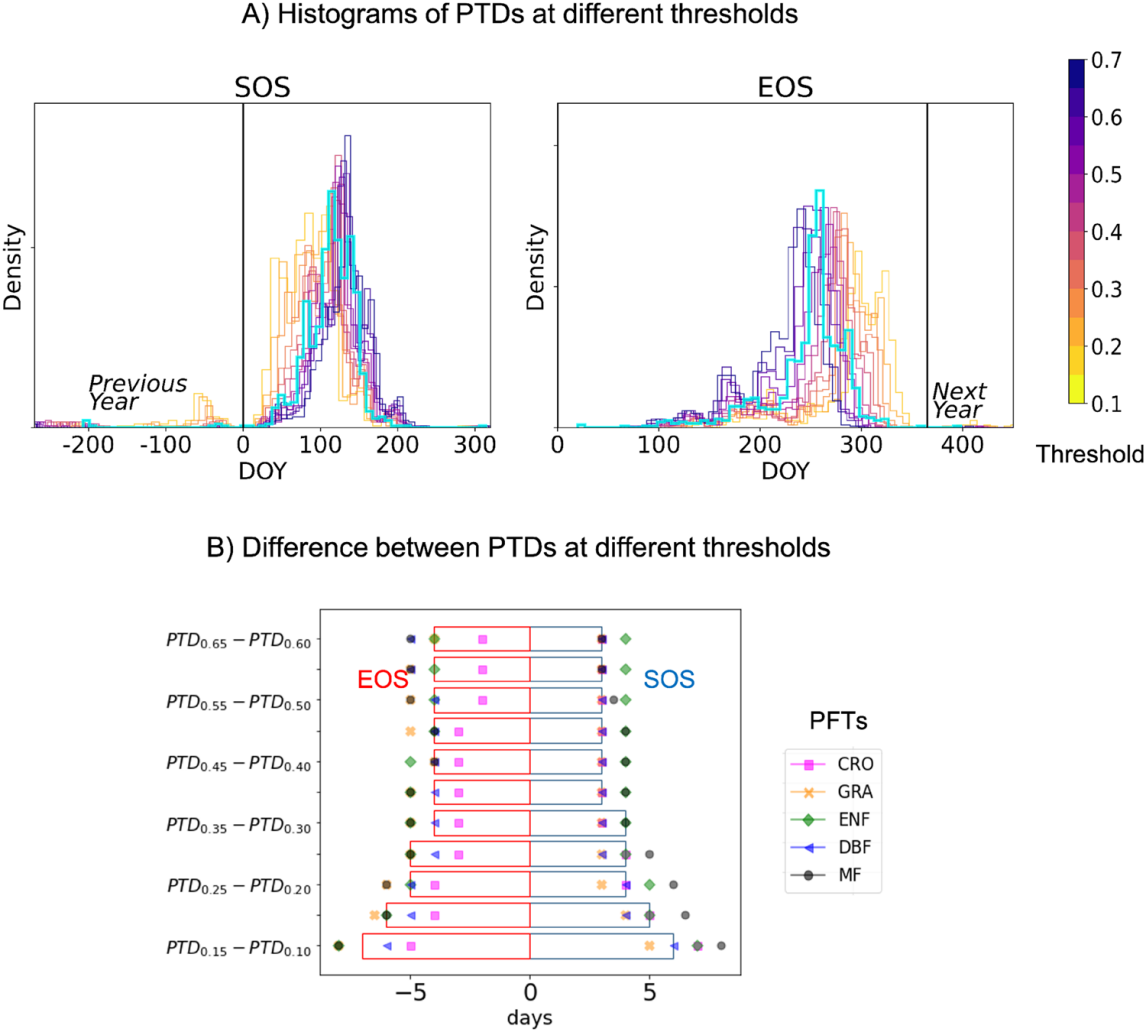
(**A**) Histograms of SOS and EOS obtained from different thresholds (see color bar) and first derivative method (cyan) (**B**)The bar plot of the median of the difference of PTDs at successive threshold values for SOS (blue) and EOS (red). Symbols show the median values for each PFTs.

Our first objective is to quantify the differences between PTDs calculated from different thresholds and derivative method. Figure 3A shows the histograms of SOS and EOS obtained at different thresholds (0.1–0.7, with an increment of 0.05) for 83 EC sites from different PFTs. Lower threshold values yield to earlier SOS, and delayed EOS. SOS and EOS obtained from 0.5 and higher thresholds are found to be closer to the one estimated from the first derivative method (in cyan). To illustrate the distinction between each thresholds. Figure 3B shows the bar plot of the differences between PTDs calculated from subsequent thresholds. In general, these differences are higher at lower thresholds and remain quite consistent ( by 3–4 days), as one moves towards the higher thresholds. When plotting the same for each PFTs, the differences among SOS are higher for evergreen needle leaf forest (ENF) at lower thresholds, representing their broader GPP seasonal curve and lower seasonal amplitude. For cropland, however, the differences are not so abrupt, indicating faster increase in GPP and their narrower GPP seasonal curve. Similar observations are important to understand that the user defined threshold values can influence interpretation of vegetation activities for different PFTs.

**Figure 4:**
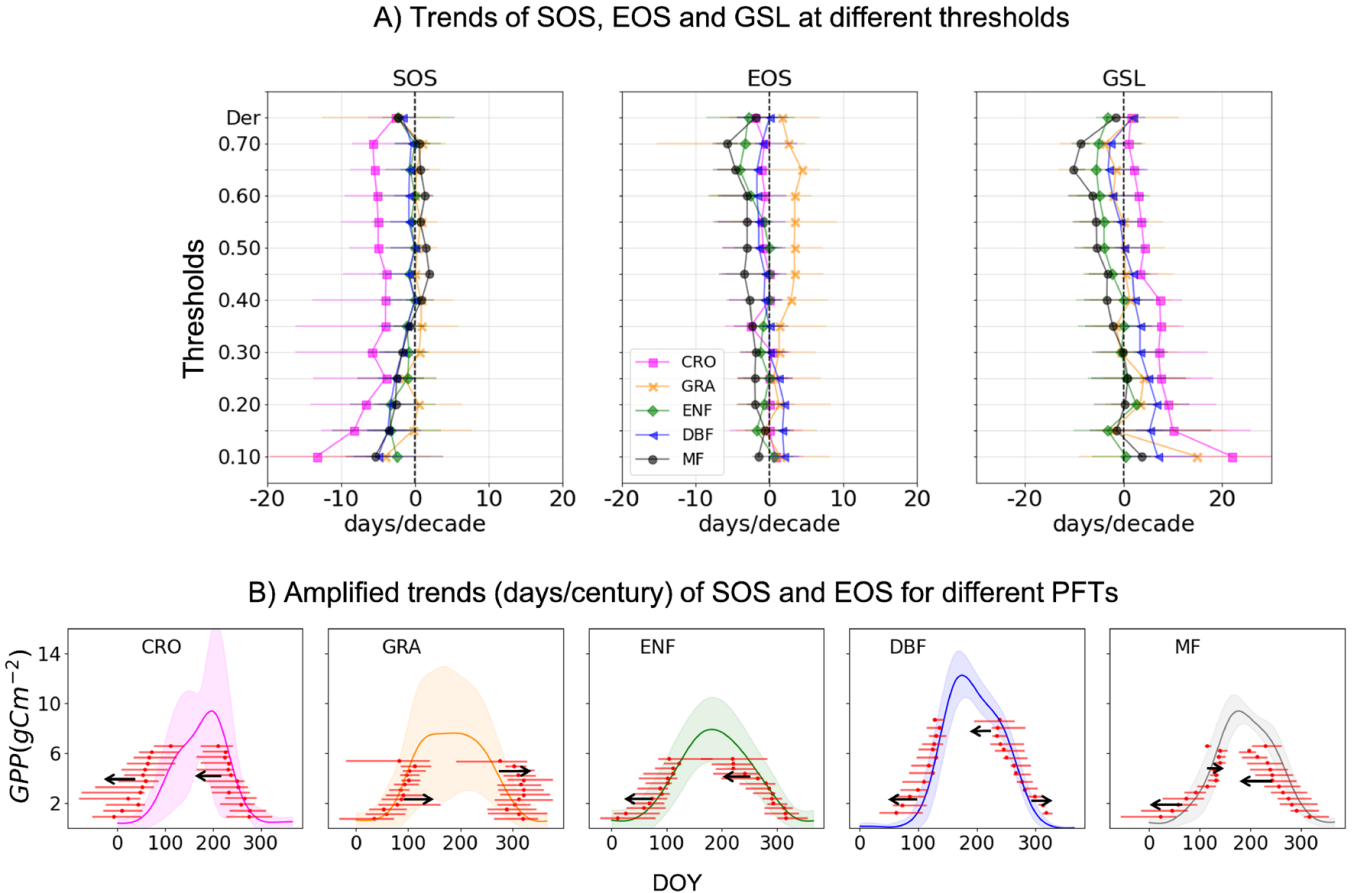
(**A**)The mean trends in SOS, EOS and GSL at different threshold values for different PFTs. (**B**) Conceptualizing the amplified SOS and EOS trends (days/century) and depiction of their impact on GPP seasonal cycle shift. The dashed lines show the median trend with their error bar representing the lower and higher confidence interval obtained from the Theil-Sen estimator. Shaded area is the standard deviation of GPP cycle for all the sites in the specific PFTs. Arrows indicate the overall direction of the shift.

The variation among PTDs calculated at different thresholds are not fully documented in the literature, and their trends can vary considerably depending on the choice of threshold. Figure 4A shows trends of SOS, EOS and GSL obtained at different thresholds and using the first derivative method for each PFTs. We found that all PFTs show advancing SOS trends at lower thresholds that tend to become weaker or even positive for mixed forest (MF) at higher thresholds. Cropland (CRO) shows the strongest early shift in SOS which is likely human induced, as earlier sowing dates are adopted to offset the impacts of climate change. Evergreen needle leaf forest (ENF), Decidous Broadleaf forest (DBF) and MF show stronger earlier shift in SOS at lower thresholds. Trends in EOS are quite distinct among PFTs. Grassland (GRA) shows delayed EOS at higher (> 0.3) thresholds. MF sites show earlier shift in EOS at all the thresholds, relatively higher negative trends towards high thresholds. For DBF, EOS trends are positive at lower thresholds and to some degree negative at higher thresholds. The contrasting trends of SOS and EOS at different thresholds also impact the trends in GSL. Overall, trends in GSL increase at lower thresholds and decrease at higher thresholds. The increasing GSL trends at lower threshold are mostly defined by the advancing trends in SOS. At higher thresholds, trends in GSL are dominantly representing the advancing trends in EOS, except for grasslands.

To evaluate how these trends impact GPP seasonal cycle, Fig. 4B shows PTDs trends along with the median GPP annual cycles for each PFTs, shaded area encloses the lower (Q1) and upper (Q3) quartile. For visualization purposes, the trends are converted into 10 times the observed trends in days/decade. Amplification of trends is not intended for forecasting but doing so it is easier to comprehend the directions of PTDs trends in relation to mean GPP seasonal cycles. Overall, except GRA all the PFTs show earlier shift in SOS but for MF, SOS shows slight delay at high thresholds. CRO, ENF and MF display clear earlier shift in EOS, in DBF however earlier EOS is only visible at higher thresholds. Only in GRA a clear delay in EOS is present notably at high thresholds. In Fig. 4 results are aggregated for each PFTs but as apparent from the high variation of GPP seasonal cycle, specially among cropland and grasslands sites, sites within same PFTs might show distinctive trends.

**Figure 5:**
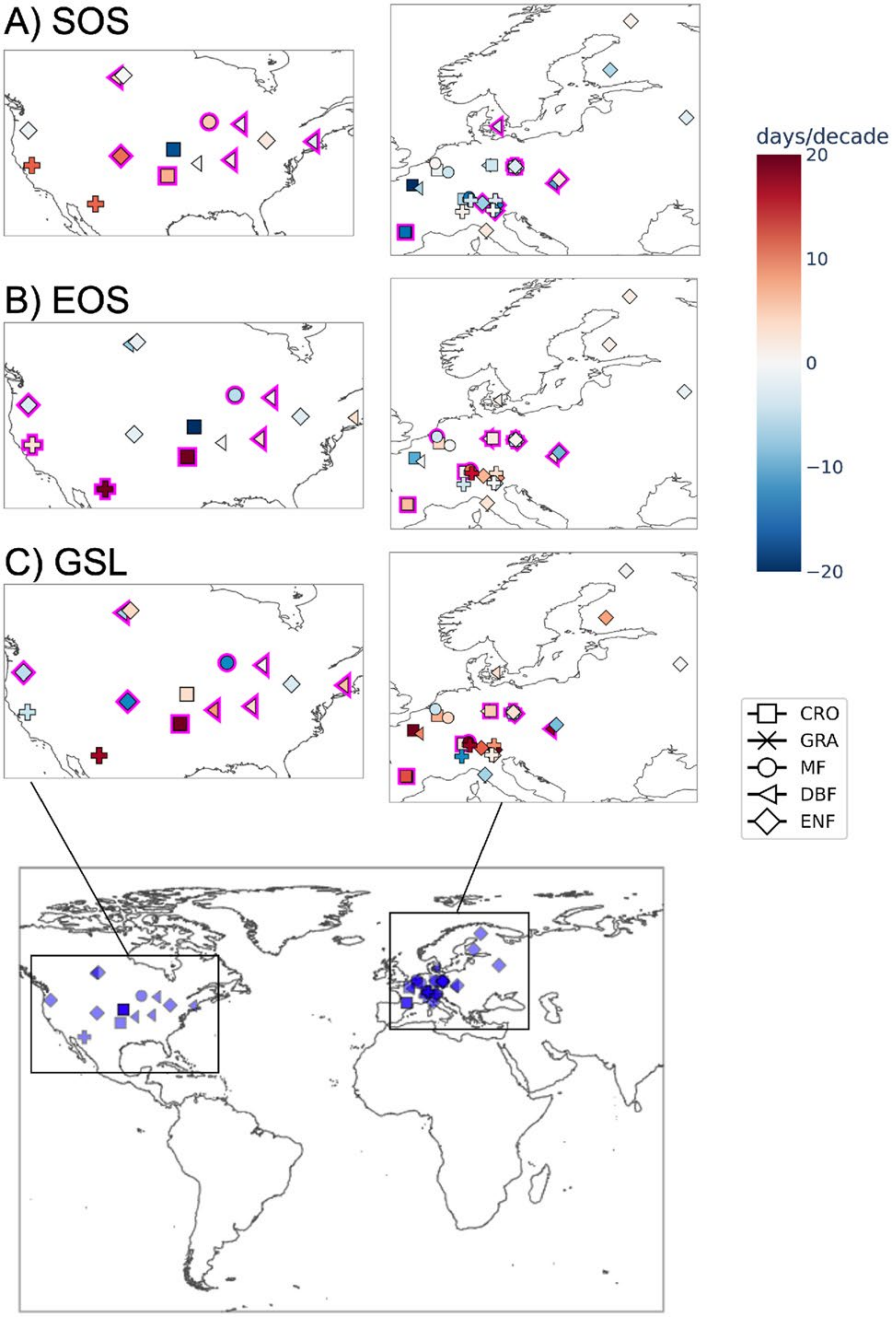
The mean trends in SOS (**A**), EOS (**B**) and GSL (**C**) at different threshold values for EC sites, see the color bar. Symbols represent different PFTs. Magenta border highlights sites with high uncertainty among trends at different thresholds. Sites with uncertain SOS and EOS trends are defined when the standard deviation is greater than 0.5 days/decade among trends at different thresholds. For GSL the uncertain sites are the sites with the standard deviation greater that 1 day/decade among trends at different thresholds.

Next, we investigate how trends vary at different thresholds for specific sites. Figure 5 shows the mean trends of SOS, EOS and GSL for EC sites located in the regions of North America and Europe. Symbols with a magenta border highlight the sites which show high standard deviation (> 0.5 days/decade for SOS and EOS, and > 0.5 days/decade for GSL) among trends obtained from different thresholds. The confidence interval for the high standard deviation is obtained from the bootstrapping method. For most of the sites, trends are strongly dependent on the threshold choice: out of 47 sites, 18 sites show uncertain trends in SOS, 20 sites in EOS and 29 sites in GSL trends when calculated using different threshold values. We did not find any strong association of uncertainty in trends to the PFTs or geographical distribution of sites. Also, the sites with uncertain trends in SOS did not always show uncertain trends in EOS. The uncertainty in trends can be due to the short length of the time series, for a signal that is prone to high inter-annual variability.

### Shifting phenology and carbon uptake

It is only reasonable to expect that shifts in phenological events influence the carbon balance of the ecosystem. In temperate ecosystems, earlier spring is associated with higher overall carbon assimilation simply because additional warmer days are available for photosynthetic activities^35^. Usually, to quantify relationship of PTDs and ecosystem productivity, analysis are performed for seasonal to annual scale. Here, we suggest an alternative approach that analyze immediate GPP changes around PTDs, which should contain dynamic information on GPP response to shifts in PTDs. To do so, we obtain long-term trends of GPP at weekly time scale. It is to pointed that we do not use weekdays for aggregating weekly GPP because when used for different years it can lead to mismatch in day of years. Instead, the weeks are redefined starting from the first day of the year, thereby each regression constitutes GPP observations from the aggregation of same days of the year.

**Figure 6:**
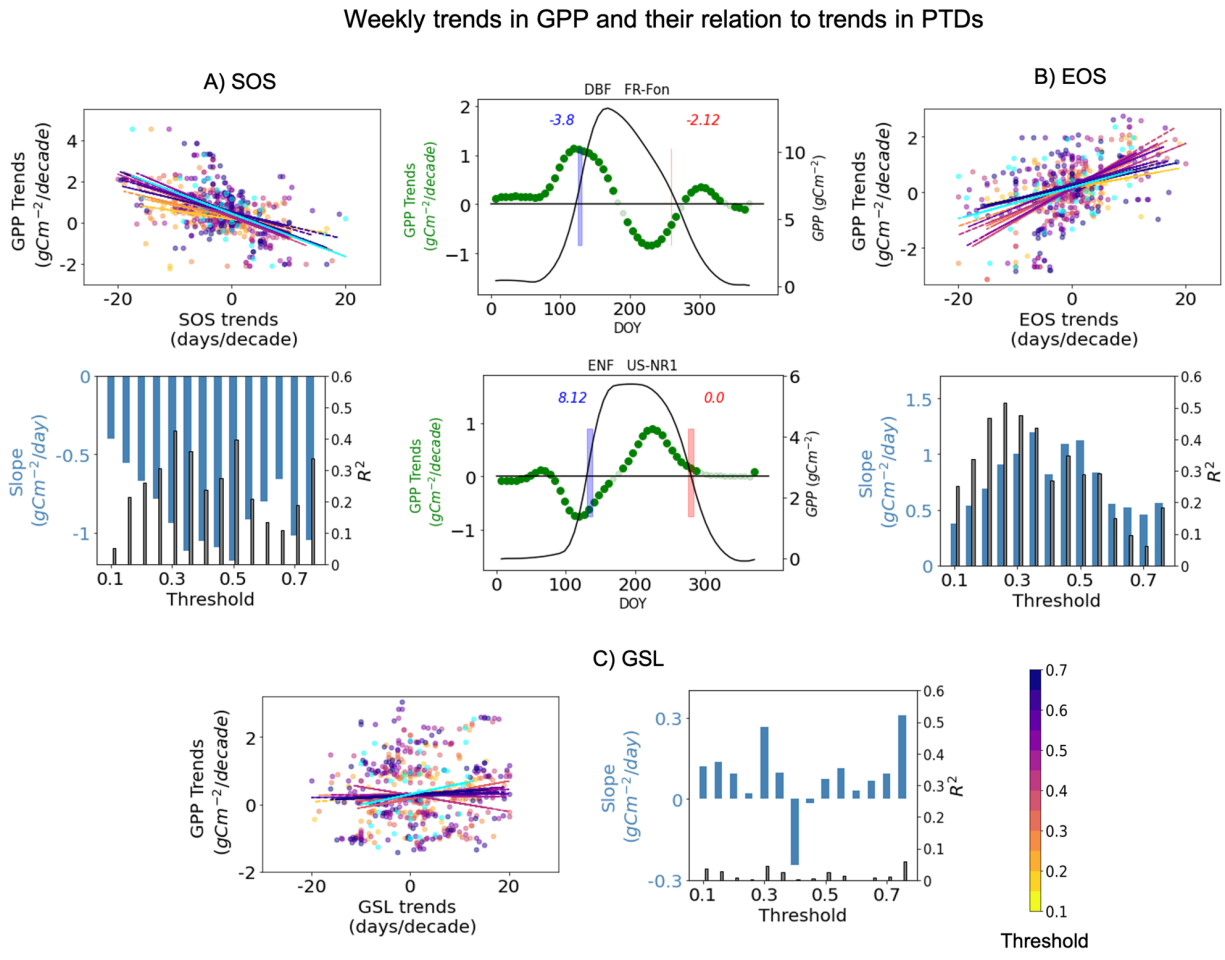
Representation of the mean trends in SOS (in blue) and EOS (in red) and weekly trends in GPP (in green, left-hand side y-axis) for two EC sites. Blue and red rectangles show the mean positions of SOS and EOS and the numbers show their mean trends in days/decade. The points with significant GPP trends are depicted in darker green color. For reference the mean annual GPP cycle is shown in black color (right-hand side y-axis). (**A**) (for SOS) and (**B**) (for EOS) show the scatter plot of PTDs trends and GPP trends around PTDs (± 2 weeks) for PTDs obtained at different threshold (see color-bar, cyan for values obtained from first derivative method). The slope shows the sensitivity of GPP trends to PTDs trends (slope), obtained from the linear regression of the scatter plots for each thresholds and $R^{2}$ shows the strength of the slope. (**C**) show similar plot for GSL and sum of GPP trends from SOS to EOS.

Figure 6 shows mean GPP annual cycle, trends in PTDs and GPP trends at weekly time-step for two EC sites. The GPP annual cycle (in black) is calculated from the daily GPP data for all the available years and is shown here as a benchmark for the interpretation of GPP trends (in green). Each green point shows GPP trend over years for the week constituting of days of years on x-axis. Darker green points being significant trends (*p* value < 0.05) and lighter green points are the non-significant trends. One can do similar analysis for daily GPP data, but daily trends can be noisy and insignificant. We choose weekly time steps because it allows more data points that is 7 times the number of years and yield robust regressions. The mean trends in SOS (in blue) and EOS (in red) are displayed in the corresponding plot, for demonstration, these trends are shown for 0.5 threshold. Position of SOS and EOS is depicted through the rectangles whose width in x-axis represents their variation along the years. In the Fr-Fon site, spring and autumn both advanced, in US-NR1 spring delayed and no trends are noticed in autumn. How representative these trends are can be assessed by the trends in weekly GPP. Clearly for site FR-Fon/US-N1 the advance/delay in spring led to increasing/decreasing trends of GPP around the mean SOS/EOS. Strikingly, advancing EOS in FR-FOn is linked to decreased GPP trends around EOS.

Figure 6 A (for SOS) and B (for EOS) summarize the relationship of PTDs trends and immediate GPP trends [± 2weeks] around the mean PTDs for all EC sites. As already stated, PTDs at different thresholds can have variable trends. On that account, the linear regression is obtained for different threshold as well as for derivative method. In general, we found that the earlier/delayed trends in SOS/EOS is well correlated to the increased productivity (GPP trends) around ( ± 2 weeks) the SOS/EOS. The bar plots show the sensitivities of GPP trends around PTDs to the trends in PTDs, they are obtained from the slopes of the linear regressions shown in the upper plots. For both SOS and EOS the slope increases with thresholds till the threshold of 0.5 and afterwards the slope reduces at higher thresholds. The $R^2$ shows the strength of the slope that is strongest for threshold values of 0.3 and 0.5 for SOS and at thresholds 0.2–0.3 for EOS. According to these analyses the GPP is highly sensitive to trends in PTDs around the mid thresholds. This is also the time when GPP increases most rapidly during the growing season. Lastly, Fig. 6C shows GSL trends and its relation to the seasonal productivity (SOS to EOS) of the ecosystem. Overall, trends in GPP are not strongly related to trends in GSL. This is also indicated via very weak $R^2$ of the slope. When comparing the results for different thresholds, mostly increase in GSL is very weakly related to increase in GPP.

## Discussion

The present study highlights the potential of eddy covariance data in examining phenological trends and the associated methodological challenges. Based on the thorough analysis of 83 EC sites we demonstrate that different smoothing and phenology detection methods can produce different phenological transition dates, and thus, user choice uncertainties must be addressed. To reduce the subjectivity in data smoothing, we presented integral smoothing method that is less sensitive to smoothing parameters in comparison to the traditional direct smoothing of GPP data. Our approach is more robust to changes in smoothing parameters, yet not fully objective. However, it is much simpler and straightforward compared to previously suggested objective approaches such as logistic function of time^36^. To further optimize smoothing parameters we recommend to use generalized cross validation methods^37^ and explore a range of smoothing parameters. In our work we only used spline smoothing, but forthcoming research can benefit by further developing integral smoothing approaches for other functional and local fitting smoothing methods. The prospective approach of integral smoothing can be easily further tested for remotely measured vegetation indices that are correlated to GPP^38,39^ signals.

This work systematically estimated the phenological changes at different thresholds and evaluated their trends and impacts on GPP fluxes. PTDs calculated at different thresholds relate to different stages of vegetation activities that are otherwise not identified when only a single threshold value is used. We show that PTDs at different thresholds are associated to the shape of the GPP seasonal cycle. For larger GSL but lower seasonal amplitude of GPP such as in ENF, the differences in PTDs are significant among lower thresholds, as depicted in Fig. 3. The seasonal cycle of GPP of croplands and DBF, on the other hand, tend to be narrow with a larger seasonal amplitude. We suggest that for multi-site analysis the optimal threshold value for PTD calculation could be a function of the width and amplitude of GPP seasonal cycles. Along these lines, a recent remote sensing study developed a regionally modified threshold algorithm to improve PTD retrievals for ecosystems with low seasonal amplitude^40^. In the present study, the threshold was prescribed for the whole growing season to retrieve SOS and EOS but previous studies have used in-season thresholds for cases when spring and autumn had different minima^41^. Similarly, in the case of asymmetric or bimodal GPP seasonal cycles, separate threshold values should be carefully selected for SOS and EOS calculations.

Our results show overall advancing trends in SOS at lower thresholds for all the PFTs except in grasslands. These findings are in agreement with the previous work based on EC sites^13^. Our analysis show weaker and uncertain EOS trends at lower thresholds, that might explain findings from the prior study^13^, where EOS was also retrieved at a lower (0.25) threshold. In certain cases for CRO, GRA and DBF, delayed EOS was observed at lower thresholds that is consistent with remote sensing findings^42–44^. However, not many studies explore trends of EOS at higher thresholds. According to our study, advancing EOS trends are prominent at higher thresholds in the majority of PFTs. Strong trends of EOS at higher thresholds are justifiable since most important climatic effect on the pigment pools degradation occur at the beginning of the autumn decay. On contrary, autumn penology at lower thresholds can be influenced by other mechanistic player such as wind induced leaf litterfall that might outclass the impact of climate change. Similarly, PTD calculated at lower thresholds are also prone to processes occurring in the understorey, that can be quite different depending on sites and site management.

Another promising finding show increasing trends in GSL at lower thresholds due to earlier shift in SOS at lower thresholds but decreasing trends at higher thresholds due to earlier shift in EOS at higher thresholds. This raises concerns about calculating GSL trends when SOS and EOS are obtained from the same threshold value. GSL trends are more indicative of ecosystem functioning when SOS is calculated from lower threshold and EOS from the higher threshold. Alternatively, GSL and its trends can be calculated for different sets of thresholds depending on the respective vegetation stage.

Even though our analysis were aggregated for different PFT types, the trends among PFTs and thresholds are still variable and uncertain for different EC sites. This may be due to the limited length of the time series of EC data and strong inter-annual fluctuations leading noise in trend analyses. To find the optimal threshold that best represents vegetation activity, integration of ground citizen phenological observations^45,46^ to EC observations could be beneficial. Moreover, complementing EC sites with digital cameras is also advocated for the objective measure of phenological stages^47,48^.

Our findings have important implications on investigating the link between PTDs trends and its influence on the ecosystem productivity. So far, previous studies focused on seasonal scale changes in phenology. For instance, based on a biophysical models and EC data, Baldocchi et al.^49^ estimated that at temperate deciduous forests one day increase in growing season length enhances the net ecosystem ${\text{CO}}_{2}$ exchange by 5.9 ${\text{gCm}}^{{ - 2}}$. Later Richardson et al.^50^ used spatial and temporal patterns of 21 FLUXNET sites to quantify the relationship between productivity and phenology through the spring time (April–June) carbon flux integrals. Several other studies also look into the individual contribution of SOS and EOS shifts on the net carbon uptake during growing season^51,52^. Following these advances, we extended GPP trend analysis for a much finer weekly scale. We found strong relationship of earlier shift in SOS/EOS to increased/decreased trends of immediate primary productivity. However, no strong relations were noted in the seasonal scale, indicating compensating effect of phenological shifts. Similar compensating effects are reported in relation to water availability and ecosystem productivity. A previous study^53^ showed that the declining net ecosystem productivity was attributed to late season summer drought induced by earlier onset of spring. For future studies, we advocate trend analysis of other confounding factors such as temperature, water and light availability to explicitly understand their individual role in shaping ecosystem productivity.

This research could provide insights into the relative importance of phenological shifts in driving seasonal scale trends in GPP. In future studies, it would be valuable to explore the individual contributions of shifting phenology on vegetation productivity in comparison to the influences of natural disturbances^29^, carbon fixation^54^ and nutrient availability^55^, which are also significant drivers of global greening trends^56,57^. By examining these factors separately, we can gain a deeper understanding of their respective impacts on vegetation productivity.

## Conclusion

Amidst surging interest in vegetation phenology, use of EC data is still limited due to underlying methodological challenges. In this work we first present an integral smoothing approach that reduces subjectivity in the smoothing of GPP time series. Moving forward, integral smoothing could be further developed for other vegetation indices. Calculating PTDs is among other key issues and till date there is no general consensus on the optimal method. Our detailed and systematic analysis on PTD calculation at different thresholds outlines reasonable concerns on using a single threshold and call for a systematic use of multiple thresholds in phenological studies from satellite, phenocam and flux observations. PTD trends can vary in magnitude or even in direction when different thresholds are used. In general, vegetation from different PFTs show stronger earlier shifts in SOS at lower thresholds. On the contrary, EOS trends were variable at lower thresholds, but showed gradually advancing trends at higher thresholds. These findings should be considered when selecting thresholds for specific ecosystems, seasons and PFTs. Within this framework we presented GPP trends at weekly scales, providing quantitative insights into the impact of PTD shifts on ecosystem productivity. Annual seasonal productivity were only weakly related to growing season length, besides, this relationship can vary with the threshold values. Our findings show that the shifts in PTDs mainly impact the immediate productivity around PTDs, while the seasonal productivity can depend on other confounding factors such as water availability and radiation. Overall, this study addresses the existing methodological challenges and present new perspectives to improve the use of EC data in measuring vegetation phenological responses to climate change.

**Table 1:** Site information of eddy covariance data used from the Fluxnet 2015, Ameriflux, and ICOS Warm Winter 2020 releases. PFT denotes the IGBP plant functional type of the site. * marks sites which were used in the trend analysis.,

Site ID (PFT)	Data source	DOI
AT-Neu* (GRA)	FLUXNET2015	https://doi.org/10.18140/FLX/1440121
AU-DaP (GRA)	FLUXNET2015	https://doi.org/10.18140/FLX/1440123
BE-Bra* (MF)	WarmWinter2020	https://doi.org/10.18160/2G60-ZHAK
BE-Lon* (CRO)	WarmWinter2020	https://doi.org/10.18160/2G60-ZHAK
BE-Vie* (MF)	WarmWinter2020	https://doi.org/10.18160/2G60-ZHAK
CA-Gro (MF)	FLUXNET2015	https://doi.org/10.18140/FLX/1440034
CA-Man (ENF)	FLUXNET2015	https://doi.org/10.18140/FLX/1440035
CA-Oas* (DBF)	FLUXNET2015	https://doi.org/10.18140/FLX/1440043
CA-Obs* (ENF)	FLUXNET2015	https://doi.org/10.18140/FLX/1440044
CA-Qfo (ENF)	FLUXNET2015	https://doi.org/10.18140/FLX/1440045
CA-TP1 (ENF)	FLUXNET2015	https://doi.org/10.18140/FLX/1440050
CA-TP3 (ENF)	FLUXNET2015	https://doi.org/10.18140/FLX/1440052
CA-TP4* (ENF)	FLUXNET2015	https://doi.org/10.18140/FLX/1440053
CH-Aws (GRA)	WarmWinter2020	https://doi.org/10.18160/2G60-ZHAK
CH-Cha* (GRA)	WarmWinter2020	https://doi.org/10.18160/2G60-ZHAK
CH-Dav* (ENF)	WarmWinter2020	https://doi.org/10.18160/2G60-ZHAK
CH-Fru* (GRA)	WarmWinter2020	https://doi.org/10.18160/2G60-ZHAK
CH-Lae* (MF)	WarmWinter2020	https://doi.org/10.18160/2G60-ZHAK
CH-Oe1 (GRA)	FLUXNET2015	https://doi.org/10.18140/FLX/1440135
CH-Oe2* (CRO)	WarmWinter2020	https://doi.org/10.18160/2G60-ZHAK
CZ-BK1* (ENF)	WarmWinter2020	https://doi.org/10.18160/2G60-ZHAK
CZ-BK2 (GRA)	FLUXNET2015	https://doi.org/10.18140/FLX/1440144
CZ-RAJ (ENF)	WarmWinter2020	https://doi.org/10.18160/2G60-ZHAK
CZ-Stn* (DBF)	WarmWinter2020	https://doi.org/10.18160/2G60-ZHAK
DE-Geb* (CRO)	WarmWinter2020	https://doi.org/10.18160/2G60-ZHAK
DE-Gri* (GRA)	WarmWinter2020	https://doi.org/10.18160/2G60-ZHAK
DE-Hai* (DBF)	WarmWinter2020	https://doi.org/10.18160/2G60-ZHAK
DE-Hzd (DBF)	WarmWinter2020	https://doi.org/10.18160/2G60-ZHAK
DE-Kli* (CRO)	WarmWinter2020	https://doi.org/10.18160/2G60-ZHAK
DE-Lnf (DBF)	FLUXNET2015	https://doi.org/10.18140/FLX/1440150
DE-Obe* (ENF)	WarmWinter2020	https://doi.org/10.18160/2G60-ZHAK
DE-RuR (GRA)	WarmWinter2020	https://doi.org/10.18160/2G60-ZHAK
DE-RuS (CRO)	WarmWinter2020	https://doi.org/10.18160/2G60-ZHAK
DE-RuW (ENF)	WarmWinter2020	https://doi.org/10.18160/2G60-ZHAK
DE-Tha* (ENF)	WarmWinter2020	https://doi.org/10.18160/2G60-ZHAK
DK-Sor* (DBF)	WarmWinter2020	https://doi.org/10.18160/2G60-ZHAK
FI-Hyy* (ENF)	WarmWinter2020	https://doi.org/10.18160/2G60-ZHAK
FI-Let (ENF)	WarmWinter2020	https://doi.org/10.18160/2G60-ZHAK
FI-Sod* (ENF)	FLUXNET2015	https://doi.org/10.18140/FLX/1440160
FR-Aur* (CRO)	WarmWinter2020	https://doi.org/10.18160/2G60-ZHAK
FR-Bil (ENF)	WarmWinter2020	https://doi.org/10.18160/2G60-ZHAK
FR-Fon* (DBF)	WarmWinter2020	https://doi.org/10.18160/2G60-ZHAK
FR-Gri* (CRO)	WarmWinter2020	https://doi.org/10.18160/2G60-ZHAK
FR-Hes (DBF)	WarmWinter2020	https://doi.org/10.18160/2G60-ZHAK
FR-LBr (ENF)	FLUXNET2015	https://doi.org/10.18140/FLX/1440163
FR-Lam* (CRO)	WarmWinter2020	https://doi.org/10.18160/2G60-ZHAK
IL-Yat* (ENF)	WarmWinter2020	https://doi.org/10.18160/2G60-ZHAK
IT-BCi (CRO)	WarmWinter2020	https://doi.org/10.18160/2G60-ZHAK
IT-Col (DBF)	FLUXNET2015	https://doi.org/10.18140/FLX/1440167
IT-Lav* (ENF)	WarmWinter2020	https://doi.org/10.18160/2G60-ZHAK
IT-MBo* (GRA)	WarmWinter2020	https://doi.org/10.18160/2G60-ZHAK
IT-Ren* (ENF)	WarmWinter2020	https://doi.org/10.18160/2G60-ZHAK
IT-Ro1 (DBF)	FLUXNET2015	https://doi.org/10.18140/FLX/1440174
IT-Ro2 (DBF)	FLUXNET2015	https://doi.org/10.18140/FLX/1440175
IT-SR2 (ENF)	WarmWinter2020	https://doi.org/10.18160/2G60-ZHAK
IT-SRo* (ENF)	FLUXNET2015	https://doi.org/10.18140/FLX/1440176
IT-Tor* (GRA)	WarmWinter2020	https://doi.org/10.18160/2G60-ZHAK
NL-Hor (GRA)	FLUXNET2015	https://doi.org/10.18140/FLX/1440177
RU-Fyo* (ENF)	WarmWinter2020	https://doi.org/10.18160/2G60-ZHAK
SE-Nor (ENF)	WarmWinter2020	https://doi.org/10.18160/2G60-ZHAK
SE-Ros (ENF)	WarmWinter2020	https://doi.org/10.18160/2G60-ZHAK
SE-Svb (ENF)	WarmWinter2020	https://doi.org/10.18160/2G60-ZHAK
US-ARM* (CRO)	AmerifluxFLUXNET	https://doi.org/10.17190/AMF/1854366
US-Blo (ENF)	FLUXNET2015	https://doi.org/10.18140/FLX/1440068
US-GLE (ENF)	FLUXNET2015	https://doi.org/10.18140/FLX/1440069
US-Ha1* (DBF)	FLUXNET2015	https://doi.org/10.18140/FLX/1440071
US-IB2 (GRA)	FLUXNET2015	https://doi.org/10.18140/FLX/1440072
US-MMS* (DBF)	AmerifluxFLUXNET	https://doi.org/10.17190/AMF/1854369
US-MOz* (DBF)	AmerifluxFLUXNET	https://doi.org/10.17190/AMF/1854370
US-Me2* (ENF)	AmerifluxFLUXNET	https://doi.org/10.17190/AMF/1854368
US-NR1* (ENF)	FLUXNET2015	https://doi.org/10.18140/FLX/1440087
US-Ne1* (CRO)	FLUXNET2015	https://doi.org/10.18140/FLX/1440084
US-Ne2* (CRO)	FLUXNET2015	https://doi.org/10.18140/FLX/1440085
US-Ne3* (CRO)	FLUXNET2015	https://doi.org/10.18140/FLX/1440086
US-Oho (DBF)	FLUXNET2015	https://doi.org/10.18140/FLX/1440088
US-PFa* (MF)	FLUXNET2015	https://doi.org/10.18140/FLX/1440089
US-SRG (GRA)	FLUXNET2015	https://doi.org/10.18140/FLX/1440114
US-Syv (MF)	FLUXNET2015	https://doi.org/10.18140/FLX/1440091
US-UMB* (DBF)	FLUXNET2015	https://doi.org/10.18140/FLX/1440093
US-UMd (DBF)	FLUXNET2015	https://doi.org/10.18140/FLX/1440101
US-Var* (GRA)	FLUXNET2015	https://doi.org/10.18140/FLX/1440094
US-WCr (DBF)	FLUXNET2015	https://doi.org/10.18140/FLX/1440095
US-Wkg* (GRA)	FLUXNET2015	https://doi.org/10.18140/FLX/1440096

## Data Availability

The datasets analysed during the current study are publicly available at FLUXNET 2015 (https://fluxnet.org/data/fluxnet2015-dataset/), ICOS warm winter 2020 (https://www.icos-cp.eu/data-products/2G60-ZHAK) and AmeriFlux FLUXNET (https://ameriflux.lbl.gov/data/aboutdata/), all of which were processed using the OneFlux processing pipeline^30^. The site information is summarized in the following table. For general analysis of Figure 2 and Figure 3, 83 EC sites are used. For the trend analysis, 47 EC sites that have more than 10 years of quality data are used. More information on data of individual sites is available at the DOIs provided in Table 1.
